# Supplementary material for: Machine Learning Models for Tracking Blood Loss and Resuscitation in a Hemorrhagic Shock Swine Injury Model
Source: Bioengineering (Basel). 2024 Oct 27;11(11):1075. doi: 10.3390/bioengineering11111075 (PMC11591271; doi:10.3390/bioengineering11111075)
Supplement: Supplementary file 1 [file bioengineering-11-01075-s001.zip › bioengineering-3269547-supplementary.pdf]

bioengineering

MDPI

Article

Machine Learning Models for Tracking Blood Loss and Resuscitation in a Hemorrhagic Shock Swine Injury Model

Jose M. Gonzalez, Ryan Ortiz, Lawrence Holland, Austin Ruiz, Evan Ross and Eric J. Snider *

Organ Support and Automation Technologies Group, U.S. Army Institute of Surgical Research, Joint Base San Antonio, Fort Sam Houston, San Antonio, TX 78234, USA; jose.m.gonzalez355.civ@health.mil (J.M.G.)
* Correspondence: eric.j.snider3.civ@health.mil; Tel.: +1-210-539-8721

Citation: Gonzalez, J.M.; Ortiz, R.; Holland, L.; Ruiz, A.; Ross, E.; Snider, E.J. Machine Learning Models for Tracking Blood Loss and Resuscitation in a Hemorrhagic Shock Swine Injury Model. *Bioengineering* **2024**, *11*, x. https://doi.org/10.3390/bioengineering11111075

Academic Editors: Yunfeng Wu

Received: 4 October 2024
Revised: 21 October 2024
Accepted: 23 October 2024
Published: 27 October 2024

Copyright: © 2024 by the authors. Submitted for possible open access publication under the terms and conditions of the Creative Commons Attribution (CC BY) license (https://creativecommons.org/licenses/by/4.0/).

Supplementary Information

Supplementary Table S1. Statistical analysis for area under the ROC metrics for each predictive model. Friedman's test post-hoc Dunn's test was used to compare differences between each model. (ns denotes not significant; * denotes p < 0.05, ** denotes p < 0.01, *** denotes p < 0.001, **** denotes p < 0.0001).

AUROC	CRM-DL	CRM-ML	BLVM	PEBL	HemArea
CRM-ML	ns				
BLVM	**	****			
PEBL	**	****	ns		
HemArea	ns	ns	*	*	
MAP	ns	ns	ns	ns	ns

Supplementary Table S2. Statistical analysis for hemorrhage prediction time metric for each predictive model. Friedman's test post-hoc Dunn's test was used to compare differences between each model. (ns denotes not significant; * denotes p < 0.05, ** denotes p < 0.01, *** denotes p < 0.001, **** denotes p < 0.0001).

Hemorrhage Prediction Time	CRM-DL	CRM-ML	BLVM	PEBL	HemArea
CRM-ML	ns				
BLVM	*	****			
PEBL	*	****	ns		
HemArea	ns	****	ns	ns	
MAP	ns	**	ns	ns	ns

Supplementary Table S3. Statistical analysis for resuscitation to hemorrhage score ratio for each predictive model. Friedman's test post-hoc Dunn's test was used to compare differences between each model. (ns denotes not significant; * denotes p < 0.05, ** denotes p < 0.01, *** denotes p < 0.001, **** denotes p < 0.0001).

Resuscitation Score Ratio	CRM-DL	CRM-ML	BLVM	PEBL	HemArea
CRM-ML	ns				
BLVM	**	***			
PEBL	ns	ns	*		
HemArea	ns	ns	**	ns	
MAP	ns	ns	ns	ns	ns

Disclaimer/Publisher's Note: The statements, opinions and data contained in all publications are solely those of the individual author(s) and contributor(s) and not of MDPI and/or the editor(s). MDPI and/or the editor(s) disclaim responsibility for any injury to people or property resulting from any ideas, methods, instructions or products referred to in the content.
